# Massachusetts Veterinary Association

**Published:** 1900-02

**Authors:** Henry D. Lewis

**Affiliations:** Secretary


### MASSACHUSETTS VETERINARY ASSOCIATION.

THE regular monthly meeting was held on the evening of December 27th at 19 Boylston Place, Boston. President Langdon Frothingham called the meeting to order at 8.15. Minutes of previous meeting were read and adopted.

Dr. John M. Parker moved that the Secretary be instructed to send a constitution and application-blank to every graduated practising veterinarian in the State. Dr. Daniel Emerson seconded the motion; carried.

The essayist for the evening, Dr. Frothingham, read the following paper on "Actinomycosis."

He reviewed the subject of actinomycosis and compared this disease with bothryomycosis, a disease of the horse manifested by an infectious tumor—bothryomycoma—which both macroscopically and microscopically closely resembles the actinomycoma. He spoke of the various animals in which actinomycosis has been observed and the portions of the body where these tumors have been found, particularly of actinomycosis of the udder in cattle. Although the disease in this organ is rather uncommon, it has been thoroughly described by several European investigators. Jensen, of Copenhagen, for instance, has written of twenty cases, and calls attention to its occurrence in two distinct forms. In one form, the more common, the lesions are usually not numerous, but are apt to be extensive, a single tumor becoming as large as a hen's egg or even larger, and containing much pus. One or more quarters of the udder may be involved. In the other form the udder may become double its normal size in the course of a few months, and is very hard and rough. Sometimes only one quarter, sometimes all four are attacked. The cut surface may seem almost normal, but upon close inspection the lobules are seen to be larger than usual, and to contain nodules with purulent centres. These nodules may be exceedingly small, hardly visible to the unaided eye, or they may be as large as a pea. They may be found in countless numbers scattered throughout the gland, and are also found on the mucous membrane of the milk-ducts and cisterna. Such cases of actinomycosis of the udder so closely resemble miliary tuberculosis of that organ that a diagnosis can only be made by the microscope. Actinomycosis of the udder in swine is mentioned as a common occurrence in Europe by Johne and others. The essayist had never seen a case in this country. He then demonstrated the udder of a cow which showed extensive lesions illustrating the second form of actinomycosis as described by Jensen. He called attention to the peculiar pus from the nodules which showed the typical yellowish granules (ray fungus or actinomyces), always present in cases of actinomycosis, and by which a diagnosis is readily made. A quantity of these granules in a small tube of alcohol were also shown. The appearance of these granules fresh from the pus and crushed under the cover-glass was demonstrated with the microscope. Sections of the udder, stained with hæmotoxylin and eosin, were shown with both high and low power of the microscope, further illustrating the appearance of the actinomycosis and the histological structure of the nodules.

The cause of the disease was then spoken of. Pictures were shown to illustrate its polymorphous form. To what group of microorganisms the actinomyces properly belongs is still an open question, the speaker said, so that the term actinomyces seems still the best to use. The best opinion agrees that it is exceedingly difficult to cultivate this organism artificially, and that experimental inoculation from animal to animal is practically impossible. The disease, therefore, can only be infectious to a very mild degree, if at all, an assumption amply upheld by experience, since markedly diseased animals may remain for years in a herd without infecting other animals. On the contrary, the disease is often observed in mild enzootic form where herds are fed on certain grasses and grains, especially, it seems, where these are grown upon reclaimed lands. The life-history of this microorganism is unknown. It may be that its life in the animal body represents only one phase, and that the most virulent form occurs in its life upon some grasses and grains.

Dr. Frothingham then spoke of "Bothryomycosis." In 1869 Bollinger gave this name to a disease of the lungs in horses, which is characterized by fibrous tumors varying in size from a small pea to a pigeon's-egg, and having larger or smaller purulent centres. He described a specific fungus as the cause, and called it zooglasa pulmonis equi. In 1884 Rivolta again described this fungus, and classed it with the actinomyces bovis. Later, in 1885–1886, Johne and Rabe further studied this organism, succeeded in cultivating it in the laboratory, and reproduced the disease by experimental inoculation. The organism is a micrococcus which in the animal body collects in clumps, and these clumps become surrounded by a zooglosa mass. Johne called the organism micrococcus ascofurmans. Besides the tumors in the lungs, this micrococcus has been shown to be the cause of numerous other tumors in the horse. The most common is the champignon or chronic scirrhus cord; also small tumors in the cutis and subcutis at any part of the body where there is pressure from the harness (region of the collar, croup, corners of mouth, etc.), tumors on stump of tail after docking, tumors in the udder, and, finally, as the basis of "fistulous withers." The macroscopical lesions are exactly similar to those of actinomycosis, even to the yellowish granules. Differential diagnosis is only possible with the microscope when it is seen that these granules are composed of numerous round masses of zooglosa and not actinomyces. Such diagnosis is essential since in several instances scirrhus cord in the horse has been caused by the actinomyces. Up to the present time bothryomycosis has only been observed in the horse.

Sections of a tumor of the cord and one of the udder of a mare were shown.

Dr. Winchester moved a vote of thanks to the essayist.

Dr. E. T. Harrington reported a case of "Colloid Cancer of the Stomach."

Dr. J. F. Winchester reported a case of "Aneurism and Embolism of Post Aorta and both Iliacs of the Right Side and the Internal of the Left." History: A chestnut gelding, aged, was subject to attacks of colic during the past three years. Lameness first manifested during the summer of 1899, which, with rest. passed away. During the fall the lameness became very severe, especially if the horse was driven faster than a walk. This would pass away in a few days. Just previous to being killed the horse could not trot half a mile without becoming very lame and in great pain. Post-mortem showed a dilatation of the posterior aorta, with an organized clot from posterior end anteriorly to where the iliacs are given off. The iliacs of the right side were dilated and contained an organized clot for about twelve inches. The internal iliac of the left side contained a clot several inches in length.

Members present: Drs. Emerson, Frothingham, Harrington, Howard, Lee, Lewis, Parker, Pierce, Stickney, Winchester, and Winslow.

HENRY D. LEWIS,
Secretary.

